# Iliofemoral Venous Thrombectomy and Angioplasty in a Patient Following Simultaneous Pancreas-Kidney Transplant: A Case Report

**DOI:** 10.7759/cureus.74845

**Published:** 2024-11-30

**Authors:** Christopher Aduwari, Jeremy Todd, Daniel Scher, Pablo Serrano Rodriguez

**Affiliations:** 1 Radiology, George Washington University School of Medicine and Health Sciences, Washington, USA; 2 Interventional Radiology, George Washington University Hospital, Washington, USA; 3 Transplant, George Washington University Hospital, Washington, USA

**Keywords:** deep vein thrombosis (dvt), end-stage renal disease (esrd), interventional radiology (ir), ivc thrombectomy, mechanical thrombectomy (mt), pancreas and kidney transplant, pancreatic graft thrombosis, percutaneous transluminal balloon angioplasty, simultaneous pancreas-kidney transplantation, venous thrombotic event

## Abstract

Simultaneous pancreas-kidney (SPK) transplantation is a recognized treatment for patients with insulin-dependent diabetes and advanced chronic kidney disease or end-stage renal disease (ESRD), offering significant survival benefits. However, it is associated with a higher risk of venous thrombosis, which can jeopardize the survival of the pancreaticoduodenal graft. This case report describes a patient with type 2 diabetes, hypertension, and ESRD who developed acute, occlusive deep vein thrombosis (DVT) involving the right common femoral, profunda femoral, and greater saphenous veins on postoperative day 1 (POD1) following a deceased donor SPK transplant, despite systemic prophylactic anticoagulation. Subsequent imaging of the transplanted pancreas revealed a nonocclusive thrombus in the splenic vein with extension into the portal vein and intermittent reversal of diastolic flow in the pancreatic arterial Y-graft. Persistent leg swelling and a nonresolving DVT prompted interventional radiology consultation on POD15, leading to successful mechanical thrombectomy and balloon angioplasty of the external iliac vein. The improved venous outflow following the thrombectomy procedure subsequently contributed to the resolution of the pancreatic graft thrombosis. This case underscores the complexity of managing post-SPK transplantation complications and highlights the role of interventional radiology in addressing persistent thrombotic events to preserve graft function and patient outcomes.

## Introduction

Simultaneous pancreas-kidney (SPK) transplantation is an established treatment for patients with insulin-dependent diabetes who also suffer from advanced chronic kidney disease or end-stage renal disease (ESRD). In the United States, nearly 90% of pancreas transplants are performed as SPK transplants, while the remainder are performed as pancreas after kidney (PAK) transplants or pancreas transplant alone (PTA) [1,2]. Patients who undergo SPK transplants have been found to have decreased mortality and improved quality of life in large parts due to the well-established survival benefits conferred by having a kidney transplant alone (KTA). Adding a pancreas either simultaneously or sequentially may be associated with an incremental survival benefit due in part to reducing glucose variability and the need for dialysis [3,4].

Even though prior studies show survival benefits after an SPK transplant, one of the major complications is venous thrombosis at the anastomotic junctures [5]. As a matter of fact, previous studies have shown an increased risk of deep venous thrombosis (DVT) following SPK transplants compared to KTA. Despite technical refinements, pancreas allograft thrombosis remains the most common nonimmunological cause of early pancreatic graft loss in SPK transplantation [6]. The etiology of thrombosis is multifactorial, but prior studies have shown that the reversal of diastolic flow in the arteries of the transplanted pancreas is highly specific for detecting graft venous thrombosis during the first 12 days after transplantation [7]. This is a concern because complications of graft thrombosis include, but are not limited to, graft pancreatitis, reperfusion injury, as well as worsening of the current hypercoagulable state. Given that these patients are at an increased risk of DVT post-op, they are typically placed on heparin thromboprophylaxis, which has been shown to yield an approximate two-fold reduction in both pancreas thrombosis and pancreas loss for SPK, PAK, and PTA [8,9].

This particular case is peculiar as it involved a postoperative thrombosis of the right common femoral vein, right profunda femoral vein, and the right greater saphenous vein in the setting of multiple days of systemic anticoagulation. This occlusion of the outflow venous vasculature most likely exacerbated the thrombosis of the portosplenic/superior mesenteric venous circuit of the graft. We, therefore, discuss the thrombectomy of this DVT using the AngioJet Thrombectomy System (Boston Scientific, Marlborough, MA), which led to interval resolution of the graft thrombosis.

## Case presentation

A 50-year-old male patient with a past medical history of type 2 diabetes mellitus, hypertension, and ESRD underwent a deceased-donor SPK transplant. The donor pancreaticoduodenal graft was implanted in the right abdomen, with the portosplenic/superior mesenteric venous circuit of the graft anastomosed to the right common iliac vein (CIV) of the recipient. A Y-graft created from donor blood vessels was used to connect the superior mesenteric artery and the splenic artery of the donor pancreas to create the arterial supply circuit. This Y-graft was anastomosed to the recipient's right external iliac artery. The donor's kidney was implanted in the left abdomen, with the renal vein anastomosed to the left external iliac vein, the renal artery anastomosed to the left external iliac artery, and the ureter anastomosed to the bladder of the recipient.

On postoperative day 1 (POD1), a postoperative ultrasound (US) of the transplanted pancreas showed patent vasculature but mild pancreatic edema without pancreatic ductal dilatation or any peripancreatic fluid collection. However, the imaging showed a loss of diastolic flow within the parenchymal arteries, with abnormally elevated resistive indices. In addition, on POD1, the postoperative US of the bilateral lower extremities showed acute, occlusive deep vein thrombosis (DVT) within the right common femoral vein, right profunda femoral vein, and the right greater saphenous vein. This finding necessitated starting the patient on a therapeutic heparin infusion: heparin/D5W 25,000 units (18 units/kg/hour) + Premix D5W 250 mL. The patient also developed atrial fibrillation with rapid ventricular response, which was adequately converted to normal sinus rhythm on POD2 with an amiodarone bolus (150 mg IV bolus) and an infusion for continual control, i.e., amiodarone/D5W 450 mg (1 mg/minute) + Premix D5W 250 mL.

**Figure 1:**
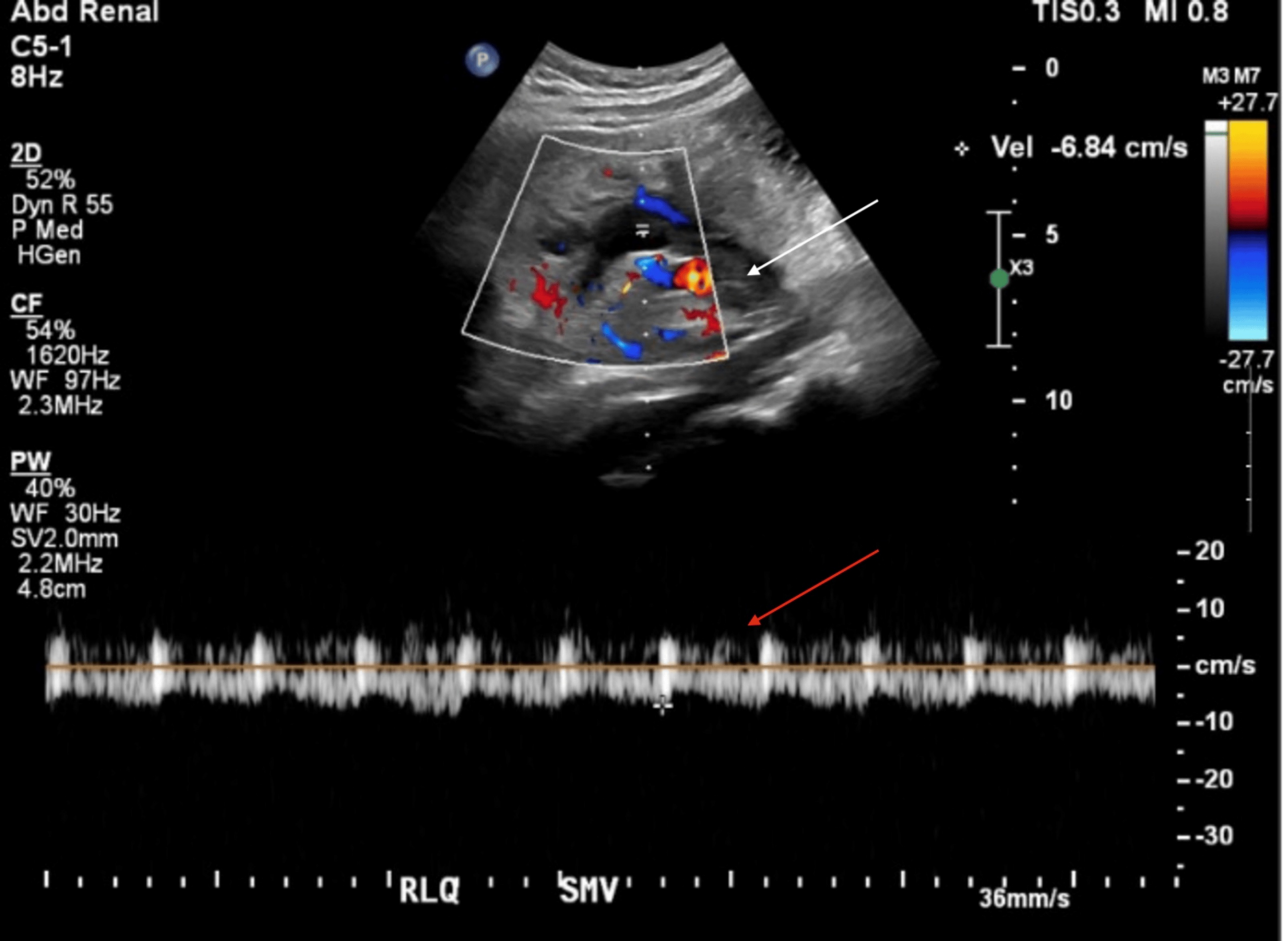
Pre-thrombectomy US with color and spectral Doppler of the transplant pancreas, demonstrating partially occlusive thrombus in the donor SMV The white arrow indicates a thrombus in the donor SMV, and the red arrow indicates a partial occlusion of the SMV, with turbulent flow visible on color Doppler and a low-velocity, relatively monophasic waveform on the spectral Doppler US: ultrasound; SMV: superior mesenteric vein

**Figure 2:**
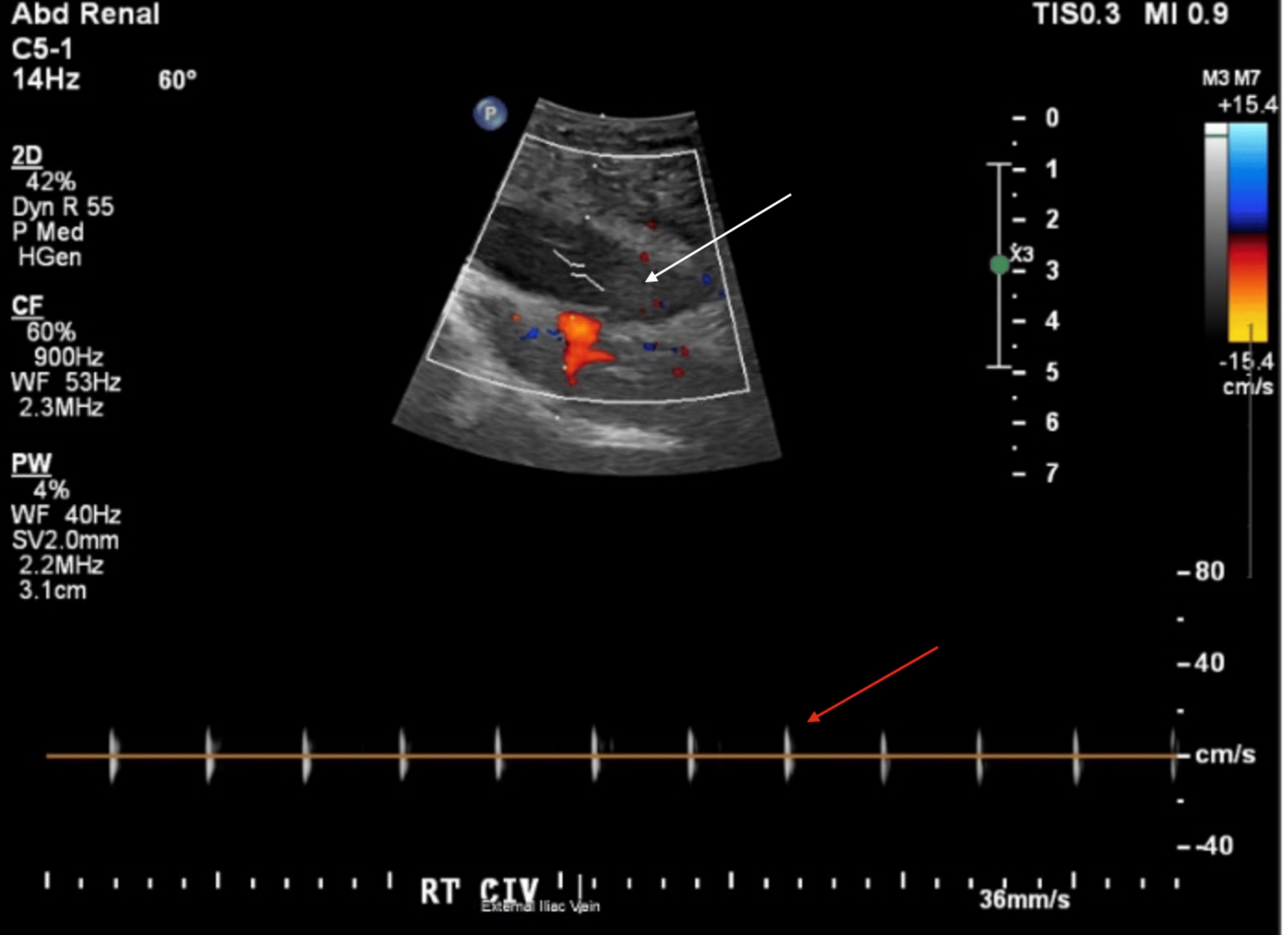
Pre-thrombectomy US with spectral and color Doppler, demonstrating occlusive thrombus in the right CIV The white arrow represents an occlusive thrombus in the right CIV, and the red arrow suggests occlusion of the right CIV, with no flow visible on the color Doppler and no fluctuations in waveform on the spectral Doppler US: ultrasound; CIV: common iliac vein

On POD4, a follow-up US of the transplanted pancreas still showed mild pancreatic edema but highlighted a new partially occlusive thrombus in the donor superior mesenteric vein (SMV) with extension into the portal vein segment, both of which remained patent (Figure 1). There was interval improvement in the resistive indices within the parenchymal arteries, although still elevated within the body and tail. Arterial flow also still remained in the pancreatic head, but there was a new intermittent reversal of diastolic flow within the Y-graft just beyond the anastomosis. Also, on POD4, the follow-up right lower extremity (RLE) US showed acute, occlusive DVT in the same vessels, unchanged from the prior scan, extending to the CIV (Figure 2). On POD15, interventional radiology was consulted for RLE thrombectomy in the setting of persistent RLE DVT following systemic anticoagulation with heparin. The goal of an indication for the thrombectomy was to improve leg swelling.

In the interventional radiology suite, left common femoral vein access was obtained with a micropuncture access set, and using the Seldinger technique, a 5-French sheath was placed to maintain access. An Omni Flush catheter (AngioDynamics, Latham, NY) was used to perform a pelvic venogram demonstrating a patent infrarenal inferior vena cava (IVC) and left iliac vein. Up and over access was obtained using the flush catheter, then the flush catheter was removed, and the 5-French sheath was appropriately up-sized to an 8-French Cook Ansel sheath (Cook Medical, Bloomington, IN).

**Figure 3:**
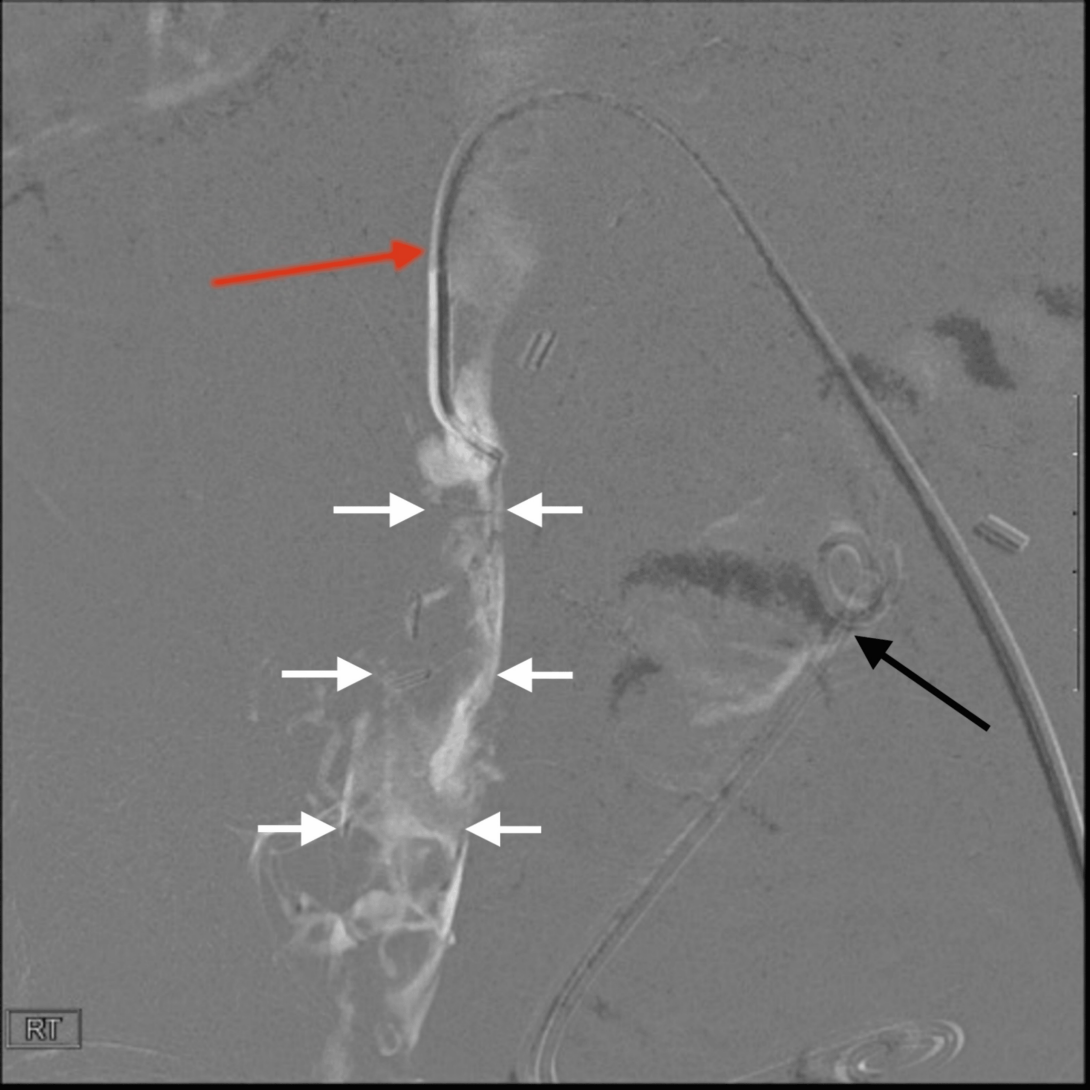
Pre-thrombectomy venogram showing nearly occlusive thrombosis in the right CIV White arrows indicate the venogram from a supine digital subtraction angiography image. The red arrow indicates that the catheter is positioned in the proximal right CIV, which shows a nearly complete occlusive thrombus. The black arrow indicates a double J stent in the left lower quadrant transplant kidney, and the ureter is also partially seen CIV: common iliac vein

**Figure 4:**
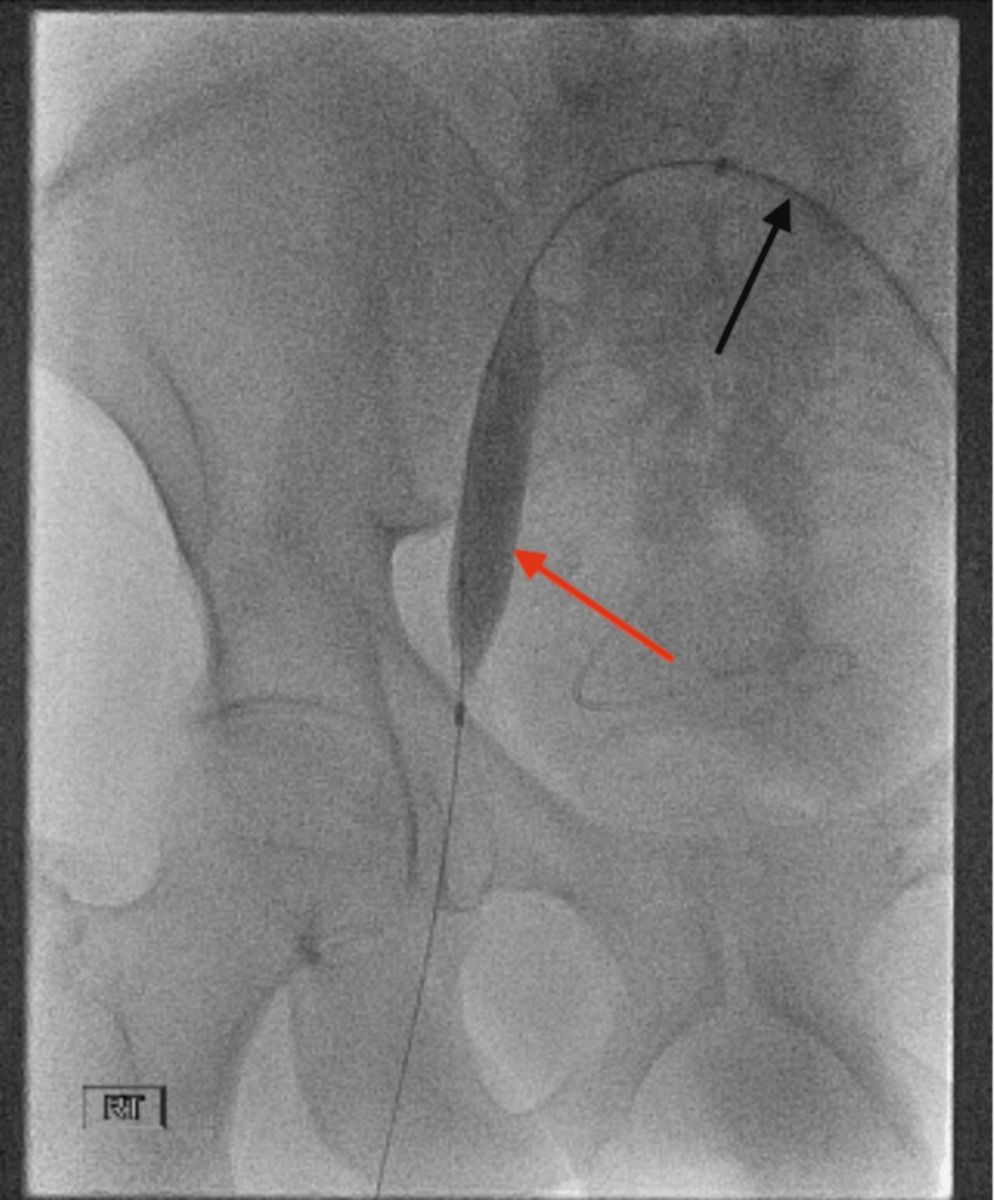
Balloon angioplasty of the right CIV This image demonstrates balloon angioplasty performed on a patient in the supine position. An 8-Fr Cook Ansel sheath (black arrow) is positioned within the left CIV, extending to the iliac vein confluence. A 14 mm × 40 mm BD Atlas angioplasty balloon (red arrow) is deployed in the right CIV and inflated to a diameter of 14 mm to restore patency CIV: common iliac vein

**Figure 5:**
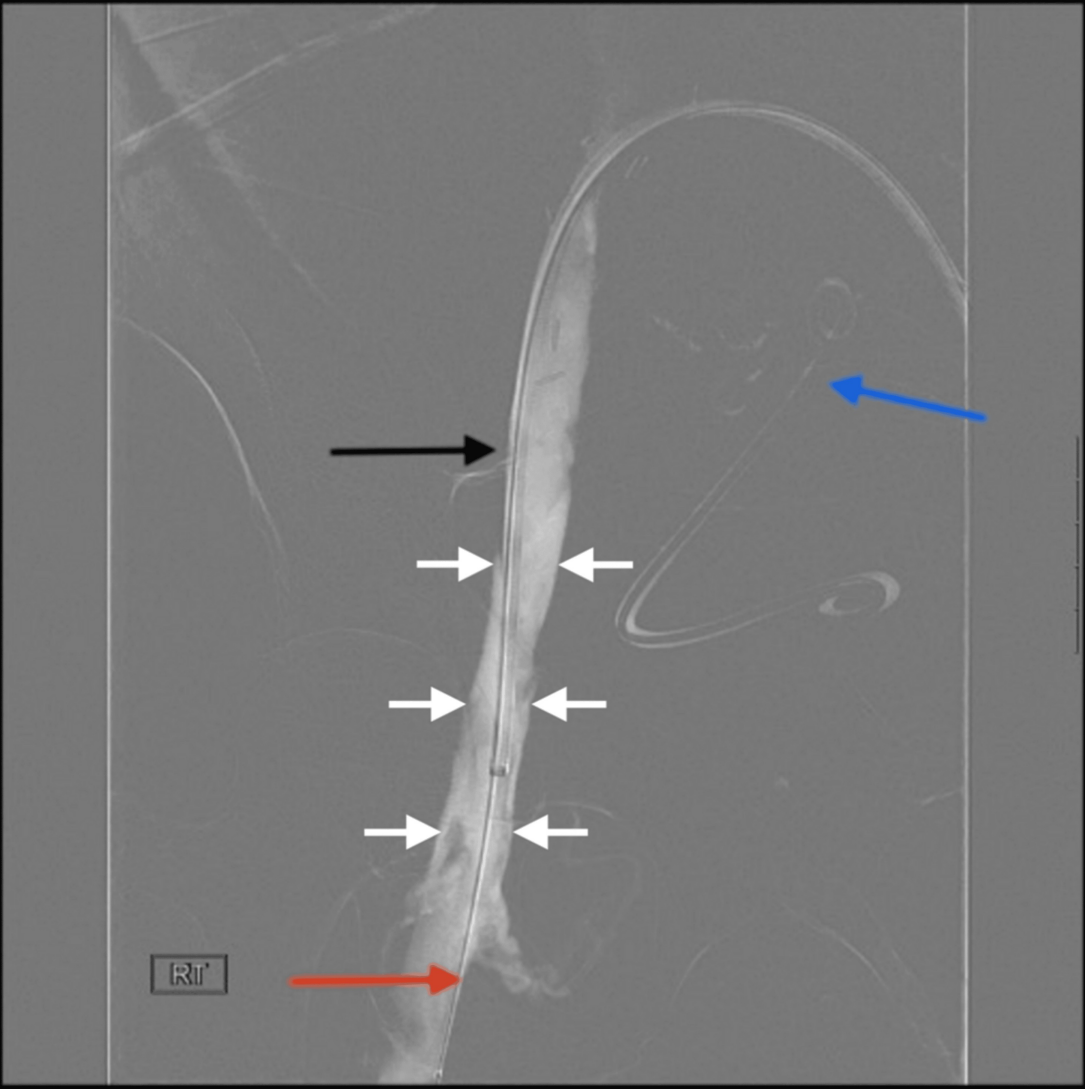
Post-thrombectomy venogram, showing patency and resolution of thrombosis in the right CIV Digital subtraction angiography image in a supine position shows a venogram; the sheath (black arrow) terminates in the distal right CIV, with the catheter (red arrow) extending off the image into the right external iliac vein. Postthrombectomy venogram demonstrates complete resolution of the previously noted thrombus in the right common iliac and right external iliac veins (white arrows). The blue arrow indicates the double J stent in the left lower quadrant transplant kidney, and the ureter is also seen CIV: common iliac vein

Access into the right common femoral vein and femoral vein was achieved using a GlideCath (Terumo, Somerset, NJ). An initial venogram from the level of the right mid-femoral vein was performed, demonstrating occlusion of the distal right external iliac vein up to the level of the IVC (Figure 3). The decision was made to perform a pharmacomechanical thrombectomy of the iliac vein using the AngioJet Thrombectomy System (Boston Scientific). Power Pulse was used first (a mode that uses a pulsatile spray mechanism to directly deliver tissue plasminogen activator into the thrombus). This was followed by thrombectomy mode. After thrombectomy, serial angioplasty was performed using a 14 mm × 40 mm BD Atlas PTA balloon (Becton, Dickinson and Company, Franklin Lakes, NJ) from the distal external iliac vein to the IVC (Figure 4). A follow-up venogram demonstrated significantly improved luminal patency of the iliac vein and near-complete resolution of the thrombus (Figure 5). There was no stent placed.

**Figure 6:**
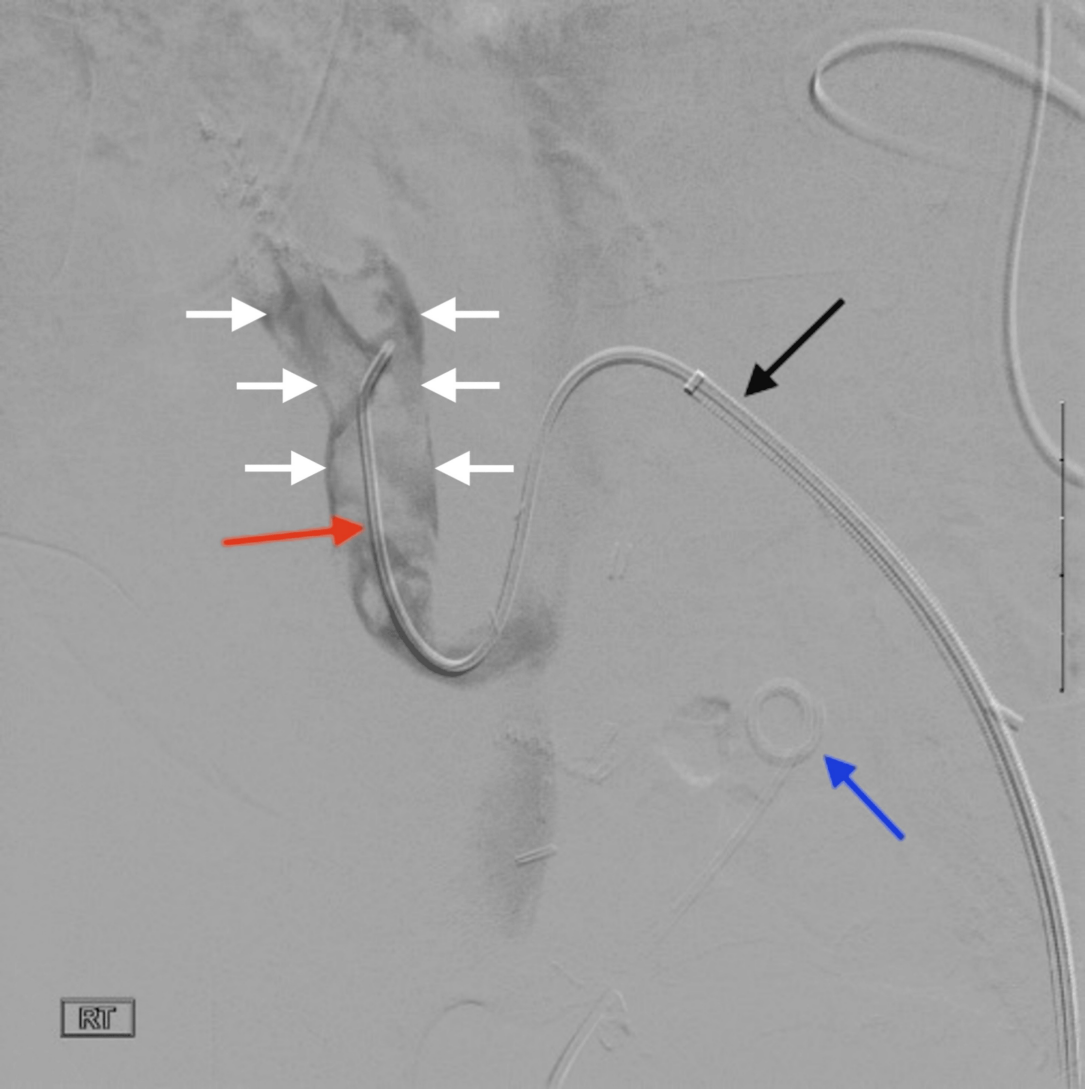
Pre-thrombectomy venogram showing nearly occlusive venous thrombosis in the pancreatic graft A venogram was performed in the supine position using digital subtraction angiography. In the image, a sheath (black arrow) is located in the left CIV. A catheter (red arrow) is also seen traversing the right CIV and connecting through a venous anastomosis between the donor's SMV and the recipient's native right CIV. The catheter terminates in the donor SMV. Notably, the donor SMV shows a nearly complete occlusive thrombus up to the level of the anastomosis (white arrows). The blue arrow indicates the double J stent in the left lower quadrant transplant kidney, and the ureter is also partially seen CIV: common iliac vein; SMV: superior mesenteric vein

**Figure 7:**
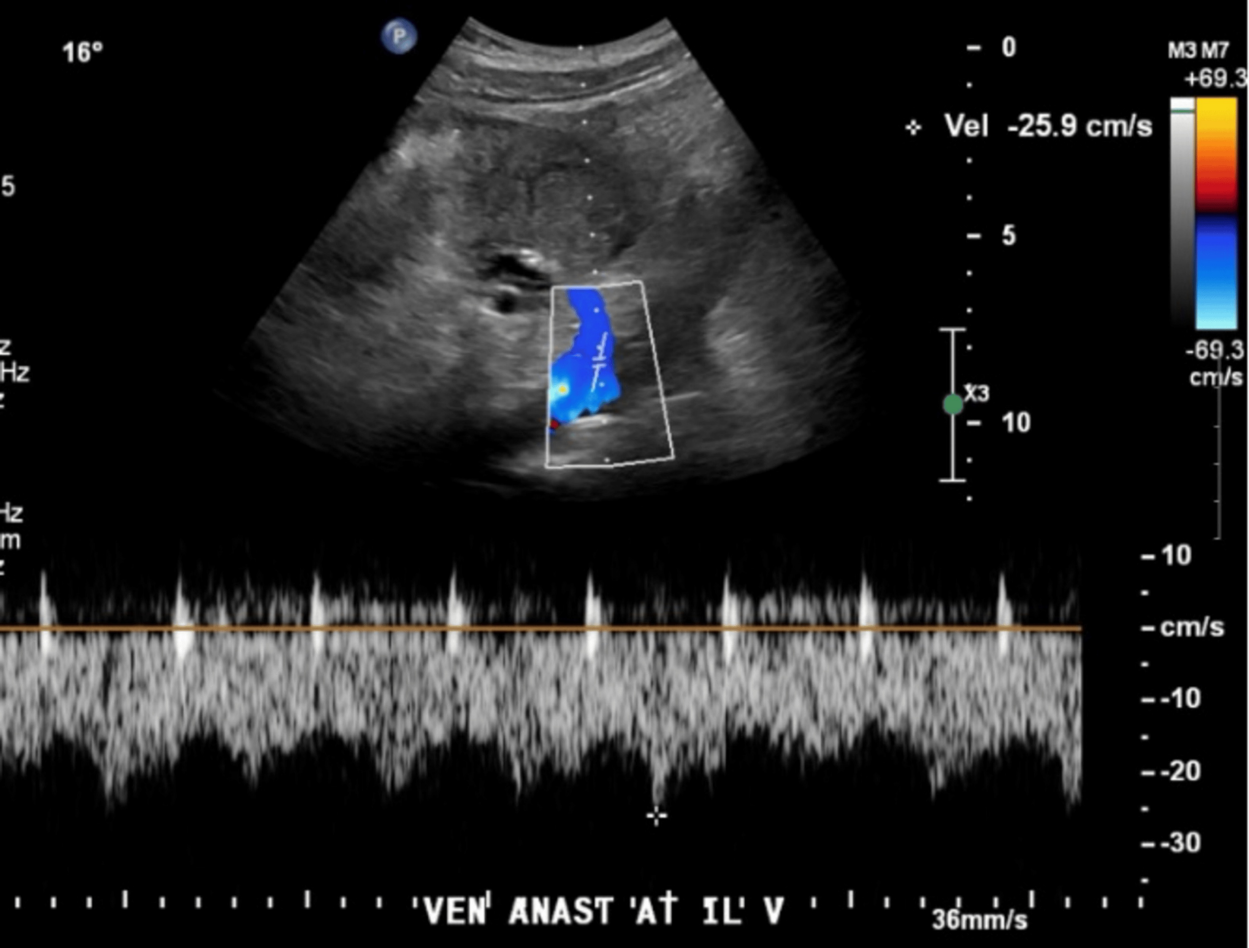
Post-thrombectomy US of the transplant pancreas, demonstrating patent donor SMV US with color and spectral Doppler imaging was performed postprocedure, revealing a patent donor SMV at the venous anastomosis site, with normal color Doppler signals and spectral Doppler waveforms US: ultrasound; SMV: superior mesenteric vein

Next, attention was directed to the portosplenic/superior mesenteric venous circuit of the pancreaticoduodenal graft to assess the venous anatomy and ensure proper access for intervention. The donor SMV was selected using the GlideCath (Terumo) and a 0.035'' wire. This technique allowed for optimal navigation and visualization of the graft's venous outflow. A venogram from the mid-distal portion of the donor SMV was performed, demonstrating multiple central luminal filling defects consistent with acute nonocclusive thrombus (Figure 6). Given the technically challenging anatomy and tortuosity of this venous circuit, a consensus was reached after a discussion with the transplant surgeon at the time to defer the thrombectomy of the transplanted pancreas. The thought process was that the thrombus should be managed conservatively to minimize possible procedural risks associated with traversing the tortuous venous anatomy. Treatment of the symptomatic DVT was the primary indication for thrombectomy, not this thrombus in the donor SMV. However, it was anticipated that a combination of relieving the downstream occlusion in addition to systemic anticoagulation would facilitate thrombus resolution without the need for super selective thrombectomy. The US of the transplanted pancreas on post-thrombectomy day 5 showed the resolution of the prior nonocclusive thrombus in the portal-splenic/superior mesenteric venous circuit (Figure 7).

## Discussion

This case highlights the complexity of managing hypercoagulability after an SPK transplant. The development of nonocclusive thrombus in the transplanted pancreatic venous circuit, despite systemic anticoagulation, aligns with previous findings that SPK transplantation is associated with a higher risk of thrombotic events compared with KTA [6]. In the study by Foshager et al. [7], various risk factors have been identified (tight anastomotic junctures, intermittent reversal of diastolic flow within the Y-graft, etc.), but no studies highlight the implication of downstream regional DVT in the exacerbation of thrombus formation.

The patient developed extensive lower extremity DVTs before the formation of the pancreatic graft thrombus. This DVT likely reduced venous outflow from the pancreatic graft, given the thrombosed vessels were located downstream from the anastomotic site. In combination with the tortuosity of the graft's venous circuit, the increased venous stasis likely exacerbated the formation of a thrombus. Initial management of the DVT involved systemic anticoagulation, as described previously. Serial readings of the activated partial thromboplastin time were maintained within the therapeutic range (51.7-87.9 seconds). Despite this, symptoms persisted, prompting a shift to a more aggressive approach. The utilization of mechanical thrombectomy and angioplasty at the downstream iliac veins eventually resolved the symptoms of the DVT while secondarily resolving the pancreatic graft thrombosis. This underscores the critical role of interventional radiology in managing these complex cases to prevent graft loss.

Although thrombectomy was not directly performed at the graft's venous circuit, the reduction in overall venous stasis was likely advantageous to venous outflow. This, in tandem with continued systemic anticoagulation, led to the resolution of the pancreatic graft thrombosis. However, this introduces the potential for recurrence of thrombosis in the future as reliance on systemic anticoagulation to resolve this issue may not always be sufficient in other patients.

The implications for clinical practice include considering early interventional radiology consultation for persistent or complicated thrombotic events. Future studies should investigate the optimal anticoagulation regimens for SPK recipients and explore whether early interventional radiology involvement could reduce the incidence of graft-threatening thrombosis. This case does not provide insights into long-term graft survival, as the focus was on the immediate postoperative period. More studies on outcomes of thrombectomy for post-SPK DVT could provide valuable information on the durability of these interventions and their impact on graft function over time.

## Conclusions

This case underscores the importance of early recognition and treatment of thrombotic complications in SPK transplant recipients. Interventional radiology played a key role in resolving the DVT and preserving graft function. As SPK transplantation continues to be a vital treatment for patients with diabetes and ESRD, the development of more tailored anticoagulation strategies and a proactive approach to managing posttransplant thrombosis will be crucial to improving patient outcomes.
